# TLR7 and TLR8 expression increases tumor cell proliferation and promotes chemoresistance in human pancreatic cancer

**DOI:** 10.3892/ijo.2015.3069

**Published:** 2015-07-02

**Authors:** TANJA GRIMMIG, NIELS MATTHES, KATHARINA HOELAND, SUDIPTA TRIPATHI, ANIL CHANDRAKER, MARTIN GRIMM, ROMANA MOENCH, EVA-MARIA MOLL, HELMUT FRIESS, IGOR TSAUR, ROMAN A. BLAHETA, CRISTOPH T. GERMER, ANA MARIA WAAGA-GASSER, MARTIN GASSER

**Affiliations:** 1 Department of Surgery I, Molecular Oncology and Immunology, University of Wuerzburg, Wuerzburg, Germany; 2 Department of Surgery I, University of Wuerzburg, Wuerzburg, Germany; 3 Brigham and Women's Hospital, Transplant Research Center, Harvard Medical School, Boston, MA, USA; 4 Department of Oral and Maxillofacial Surgery, University Hospital Tuebingen, Tuebingen, Germany; 5 Department of Surgery, University of Munich, Klinikum rechts der Isar, Munich, Germany; 6 Department of Urology, University of Frankfurt, Frankfurt am Main, Germany

**Keywords:** TLR7, TLR8, pancreatic cancer, inflammation, agonists

## Abstract

Chronic inflammation as an important epigenetic and environmental factor for putative tumorigenesis and tumor progression may be associated with specific activation of Toll-like receptors (TLR). Recently, carcinogenesis has been suggested to be dependent on TLR7 signaling. In the present study, we determined the role of both TLR7 and TLR8 expression and signaling in tumor cell proliferation and chemoresistance in pancreatic cancer. Expression of TLR7/TLR8 in UICC stage I–IV pancreatic cancer, chronic pancreatitis, normal pancreatic tissue and human pancreatic (PANC1) cancer cell line was examined. For *in vitro*/*in vivo* studies TLR7/TLR8 overexpressing PANC1 cell lines were generated and analyzed for effects of (un-)stimulated TLR expression on tumor cell proliferation and chemoresistance. TLR expression was increased in pancreatic cancer, with stage-dependent upregulation in advanced tumors, compared to earlier stages and chronic pancreatitis. Stimulation of TLR7/TLR8 overexpressing PANC1 cells resulted in elevated NF-κB and COX-2 expression, increased cancer cell proliferation and reduced chemosensitivity. More importantly, TLR7/TLR8 expression increased tumor growth *in vivo*. Our data demonstrate a stage-dependent upregulation of both TLR7 and TLR8 expression in pancreatic cancer. Functional analysis in human pancreatic cancer cells point to a significant role of both TLRs in chronic inflammation-mediated TLR7/TLR8 signaling leading to tumor cell proliferation and chemoresistance.

## Introduction

Pancreatic ductal adenocarcinoma is still an unresolved therapeutic challenge with nearly similar incidence and mortality rates. It is the most lethal type of digestive cancer with an extremely poor prognosis with a 5-year survival rate of less than 5%. Pancreatic ductal adenocarcinoma represents the fourth commonest cause of cancer related deaths and its incidence is rising in most countries (1). The only potentially curative therapy for pancreatic cancer is surgical resection. Unfortunately, only 20% of the patients have resectable cancers at the time of the diagnosis. Even among those patients who undergo resection, the 5-year survival rate is 10–25% (2,3). Preclinical and epidemiologic studies suggest inflammation as a central mediator of the neoplastic process and a potential driver of pancreatic carcinogenesis (4,5). Under-pinning this view, activation of the central signaling module of innate immunity, NF-κB has been linked to the progression of tumors (6,7); in this line, tumor immunotherapies could involve strategies that block activation of innate immune responses. On the other hand, activation of innate immunity is achieved through stimulation of pattern recognition receptors (PRRs) (8–10). Amongst these, Toll-like receptors (TLRs) were the first group to be identified. TLRs can be activated by a panel of pathogen-associated molecular patterns (PAMPs) including cell-wall components like lipopolysaccharide (LPS) as well as by microbial DNA and RNA (11). Additionally, damage-associated molecular patterns (DAMPs) which arise from inflammation and cellular injury and can stimulate TLRs and subsequently induce TLR signaling (12). Recently, enhanced expression of TLRs has been described in a variety of different tumors (13). TLRs with their ligands induces recruitment of the adapter molecule MyD88 (myeloid differentiation primary response protein 88), leading to activation of the NF-κB and MAPK-signaling pathways initiating the target products that prevent cell death by expressing anti-apoptotic proteins such as Bcl-2 and induce chronic inflammation by producing COX-2 (cyclooxygenase-2) (13,14). COX-2 together with TLR expression plays a crucial role in transformation of normal cells to cancer cells and in angiogenesis, reduced apoptosis and immunosuppression of malignant tumors (15). Our previous study indicated that endosomally expressed TLR7 and TLR8 are associated with tumor progression in colorectal cancer and reduced tumor-specific survival amongst patients with high TLR7 and TLR8 expression in colorectal cancer cells (13). In addition, some research results suggest that enforcement of innate immunity by targeted TLR activation has beneficial effects to combat tumor growth, like TLR7 agonist imiquimod, licensed for therapy of basal cell carcinoma. Other synthetic TLR7 and TLR8 agonists such as resiquimod (R848) have been developed. R848 is a selective ligand for murine TLR7 and for TLR7 and TLR8 in humans (16,17).

In the present study, we analyzed the expression of TLR7, TLR8, NF-κB and COX-2 in pancreatic cancer at different UICC stages and compared with chronic pancreatitis and healthy controls. To determine the functional role of TLR7 and TLR8 we generated TLR7 and TLR8 expressing human PANC1 cancer cells and analyzed the effects of TLR7/8 agonists (R848, resiquimod) in the inflammatory process on tumor cell proliferation and chemoresistance.

## Materials and methods

### Patients and human tissue

In a retrospective analysis, 48 out of 112 patients with a mean age of 69±5.2 years and histologically confirmed pancreatic cancer of the exocrine pancreas were evaluated in the present study. We examined only consecutive patients from which appropriate tumor material for further analysis (tumor border and tumor center) was available in a period from 06/2003 to 05/2005 in our Surgical Department approved by the local ethics committee. Patients were followed up in our Comprehensive Cancer Center (completeness index 0.96). The classification of pancreatic cancer was asserted in criterion of the Union Internationale Contre le Cancer (UICC) for determination of the tumor stage. Cancer specimens were instantly acquired in liquid nitrogen and stored at −80°C until analyzed. Tumors were evaluated for localization, tumor stage, and their differentiation grade in our Institute of Pathology.

In 69% (n=33/48) of the investigated cases, the tumor was detected in the head of the pancreas, in the remaining cases the cancer was diagnosed in the corpus or tail of the pancreas (19%, n=9/48) or in the head and corpus/tail (2%, n=1/48). We compared in a subanalysis tumor samples of UICC stage I/II (n=12) and UICC stage III/IV (n=12) patients with specimens from individuals operated on histologically confirmed chronic pancreatitis (n=8) and normal tissue of healthy controls (n=8). Paraffin sections (5 μm) were stained with haematoxylin and eosin (H&E) to assess morphology and eosinophilic areas. To determine eosinophilic areas suspicious for potential viral inclusion bodies as causative for TLR7 and TLR8 expression within the tumor we performed additional Phloxine-tartrazine staining.

### Animals

Female Balb/c nude mice were purchased from Harlan Laboratories (Rossdorf, Germany) and maintained under defined conditions in accordance with institutional guidelines from the University of Wuerzburg in Germany and the experiments were performed according to approved experimental protocols. For *in vivo* growth studies 2×10^6^ transduced PANC1 cells (TLR7^+^ PANC1, n=5; TLR8^+^ PANC1, n=5; empty vector PANC1, n=4) were injected subcutaneously into both flanks of recipient Balb/c nude mice. Mice were sacrificed (day 40) and the tumor volume was determined (V=π/6 × a × b × c, where a is the length, b is the width and c is the height).

### Immunofluorescence and immunohistochemistry

The TLR7 antibody was purchased from Imgenex Corp., (San Diego, CA, USA), the TLR8 antibody was provided by ProSci Inc. (Poway, CA, USA). COX-2 antibody was purchased from Santa Cruz Biotechnology (Santa Cruz, CA, USA) and CD34 antibody from Serotec (Duesseldorf, Germany). Isotype control antibodies were purchased by eBioscience (San Diego, CA, USA). Secondary antibodies were Cy3-conjugated AffiniPure Donkey anti-rabbit IgG (Jackson ImmunoResearch Laboratories Inc., Suffolk, UK) and Cy5-conjugated AffiniPure Donkey anti-mouse IgG. The staining was performed on serial cryostat sections of the snap-frozen specimens of pancreatic cancers (UICC II and III) with neighbouring normal pancreas (tumor border) and compared with sections from chronic pancreatitis and normal pancreas. For nuclear counterstaining slides were treated with DAPI (4′,6-Diamidino-2-phenylindoledihydrochlorid) (Sigma-Aldrich, Steinheim, Germany) or haemalaun (Sigma-Aldrich).

### Western blot analysis

Proteins were extracted from tissue samples (250 μg) using lysis buffer CytoBuster (Merck, Darmstadt, Germany) and QIAshredder (Qiagen, Hilden, Germany). Normal tissue (protein lysate) was purchased from BioChain Institute Inc. (Hayward, CA, USA). Protein samples (50 μg) were resolved by SDS-PAGE and then transferred to polyvinylidene difluoride (PVDF) membranes (Invitrogen, Carlsbad, CA, USA). Blots were probed with antibodies to TLR7 (ProSci), TLR8 (ProSci), β-actin (Santa Cruz Biotechnology) and COX-2 (Santa Cruz Biotechnology and Novus Biologicals LLC, Littleton, CO, USA). Anti-mouse IgG and anti-rabbit IgG secondary antibodies were obtained from Amersham (Braunschweig, Germany) and anti-goat IgG was purchased from Santa Cruz Biotechnology.

### FACS analysis

Cells derived from normal pancreas, chronic pancreatitis and pancreatic cancer tissues were analyzed on a flow cytometer (Beckman Coulter, Krefeld, Germany) with a software package (Coulter, Epics XL-MCL, System II). TLR7 antibody was purchased from Imgenex, TLR8 was provided by ProSci. CD34-PE antibody, FITC-conjugated anti-rabbit secondary antibody and isotype control antibodies were purchased by Beckman Coulter. For intracellular staining we used IntraPrep kit (Beckman Coulter).

### Cell culture

The human pancreatic cancer cell line PANC1 was purchased from the American Type Culture Collection (ATCC; Manassas, VA, USA) cultured in Dulbecco's modified Eagle's medium with 10% fetal bovine serum, 1% G418 and 1% penicillin/streptomycin and incubated in 5% CO_2_ at 37°C.

In contrast to tumor tissues from patients with pancreatic cancer or from patients with pancreatitis tumor cell lines express only very low levels of TLR7 and TLR8. For further studies it was necessary to overexpress both receptors in those cells. We chose PANC1, the most common established pancreatic cell line. The lentiviral transduction of TLR7 and TLR8 PANC1 cells was performed by Sirion Biotech GmbH (Martinsried, Germany). Cells were then subjected to antibiotic selection of G418-resistant cells.

### Quantitative real-time RT-PCR

Gene expression for TLR7 and TLR8 in pancreatic cancer was determined using quantitative real-time PCR (RT-qPCR). Human pancreatic matched cDNA for comparison was purchased from Pharmingen (Heidelberg, Germany) and used as control. Gene expression analyzed in pancreatic cancers was compared with normal tissue of healthy controls (n=8), chronic pancreatitis (n=8). Total cellular RNA was extracted using RNeasy Mini kit (Qiagen) according to the manufacturer's instructions. Complementary DNA (cDNA) was performed using the ImProm-II reverse transcriptase system (Promega, Mannheim, Germany) and Eppendorf Mastercycler (Eppendorf, Hamburg, Germany). TLR7 and TLR8 specific primer sets from Qiagen were used. Housekeeping gene glyceraldehyde-3-phosphate dehydrogenase (GAPDH) was used for relative quantification. PCR reactions were carried out with a DNA Engine Opticon 2 System (MJ Research; Biozym, Oldendorf, Germany).

For the experiments performed with the human pancreatic cancer cell line PANC1 gene quantification was performed with TaqMan Gene Expression Master Mix (Life Technologies, Carlsbad, CA, USA) and TaqMan Gene Expression Assays (Life Technologies) according to the manufacturer's instructions. Housekeeping genes β-actin, GAPDH, GUSB and HPRT1 were used for relative quantification. For analysis of PANC1 cells all PCR reactions were carried out with a Bio-Rad CFX96 Touch Real-Time PCR detection system.

Reproducibility was confirmed by three independent PCR runs. The relative quantification value, fold difference, is expressed as 2^−ΔΔCq^.

### Determination of the median lethal dose (LD_50_) for 5-fluorouracil

Empty vector PANC1 cells were cultured at a concentration of 5×10^3^ cells/well in 96-well plates. The cells were incubated for 48 h with 5-fluorouracil (5-FU, working concentration, 10–10,000 μmol/l; Medac, Wedel, Germany). After medium change and further 24 h at 37°C in 5% CO_2_ CellTiter 96 AQueous One Solution Cell Proliferation Assay (Promega) was performed according to the manufacturer's instructions. The median lethal dose LD_50_ was defined as amount of drug resulting in 50% killing within 2 days.

### Proliferation and resistance to chemotherapy assay

To investigate the effect of stimulation with R848 on tumor cell proliferation 2×10^6^ PANC1 cells were seeded in cell culture flasks, pre-incubated for 24 h following daily stimulation with 10 μg/ml R848 (InvivoGen, San Diego, CA, USA) for 3 days. Afterwards cells were detached and seeded 6,000 cells/well in 96-well plates. After additional incubation time of 24 and 72 h cell proliferation assay was performed as described above.

Then, we analyzed the effect of previous stimulation with R848 on the chemosensitivity of transduced PANC1 cells. Four thousand cells/well were seeded in 96-well plates, pre-incubated for 48 h and then treated with R848 (10 μg/ml). After an additional incubation of 48 h cells were treated with 500 μmol/l 5-FU and after another 48-h proliferation assay was performed as described above.

### Statistical analysis

Results were expressed as mean ± SEM in groups of patients with normal pancreatic tissue, chronic pancreatitis and pancreatic cancer. Comparisons were performed by ANOVA or paired and unpaired t-test when appropriate. Bonferroni's correction for multiple comparisons was used to determine the level of significance; P<0.05 was considered significant.

## Results

### TLR7 and TLR8 are expressed in pancreatic cancer

**Figure 1:**
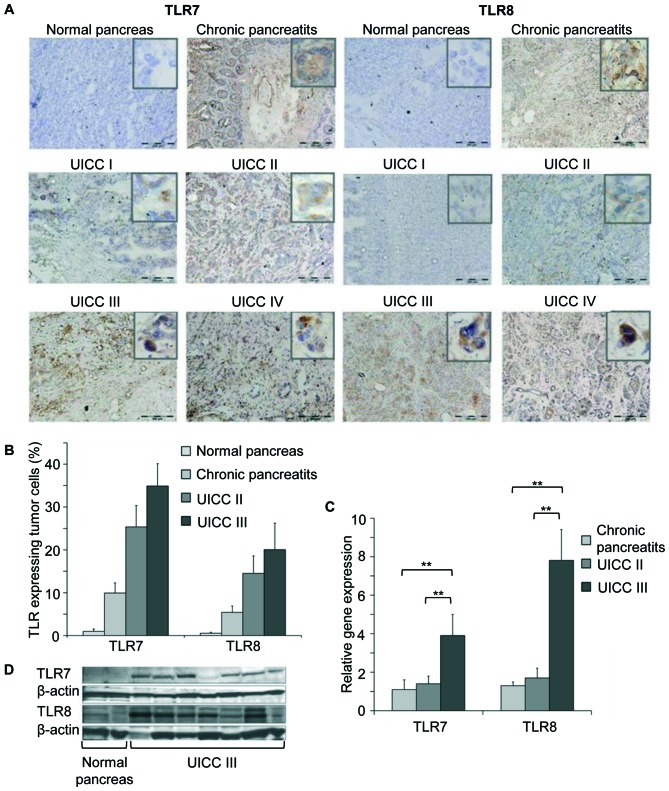
Detection of TLR7 and TLR8 expression in pancreatic cancer, chronic pancreatitis and normal pancreatic tissue. (A) In immunohistochemical staining, strong expression (UICC II) and very strong expression of TLR7 and TLR8 (UICC III) was observed in pancreatic cancer. Increased expression of TLR7 and TLR8 was detected in chronic pancreatitis. No or occasionally low expression in normal pancreas was observed. DAB (3,3′-diaminobenzidine) brown color, Haemalaun blue color for nuclear counterstaining. Original magnification, ×100 and ×200. (B) Immunohistochemical expression of TLR7 and TLR8 in normal pancreatic tissue, chronic pancreatitis, pancreatic cancer from UICC II and UICC III patients. All error bars of immunohistochemical results represent standard error of the mean. (C) Significant gene expression of TLR7 and TLR8 in advanced tumor stages (UICC III, ^**^P<0.001). Increased gene expression of TLR7 and TLR8 in low tumor stages (UICC II) and chronic pancreatitis. Normal pancreatic tissue was standardized to baseline. The relative gene expression is expressed as 2^−ΔΔCq^. (D) Confirmation of increased TLR7 and TLR8 protein expression in pancreatic cancer (UICC III) compared to normal pancreatic tissue by western blot analysis. β-actin probe was used as a control for protein loading.

TLR7 and TLR8 expression in pancreatic cancer, chronic pancreatitis, and normal pancreatic tissue was analyzed by immunohistochemistry in pancreatic tissue from patients with pancreatic cancer (UICC II and UICC III, n=48), chronic pancreatitis (n=8) and in normal pancreas (n=8). In general, TLR7 expression of pancreatic cells in all analyzed subjects with pancreatic cancer and with chronic pancreatitis was more intense than TLR8. Fig. 1A shows examples of positive TLR7 and TLR8 tumor cell expression in pancreatic cancer of different stages and chronic pancreatitis. In contrast, no or only occasionally low TLR7 or TLR8 expression was detected in normal pancreatic cells (Fig. 1A), an observation that we believe to be novel. Quantification of TLR expressing cells also demonstrated strong expression of TLR7 or TLR8 in pancreatic cells from patients with chronic pancreatitis and pancreatic cancer, compared to no, or occasionally low expression in normal pancreatic tissue (Fig. 1B). Notably, similar results were also observed by western blot analysis and gene analysis of the tumor tissues. Significant TLR7 and TLR8 protein and gene expression was observed in tissues from patients with pancreatic cancer (UICC III) compared with normal pancreatic tissue (Fig. 1C and D, respectively). These observations indicated inflammation within the tumor, which could be mediated not only through infiltrating inflammatory cells but also through TLR7 and TLR8 expression of pancreatic cancer cells.

**Figure 2:**
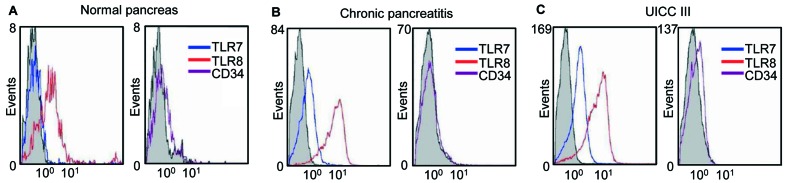
Detection of TLR7 and TLR8 expression in dissociated pancreatic cancer cells, chronic pancreatitis and normal pancreatic cells by FACS analysis. (A) In normal pancreatic cells no positivity for TLR7 and CD34 was shown. Little expression of TLR8 was observed. (B) Cells derived from chronic pancreatitis showed increased expression of TLR7 and TLR8 but not of CD34. (C) In pancreatic cancer cells (UICC III) elevated expression levels of TLR7, TLR8 and CD34 were demonstrated. TLR7 blue line, TLR8 red line, CD34 purple line and IgG control black line. One representative experiment of three.

**Figure 3:**
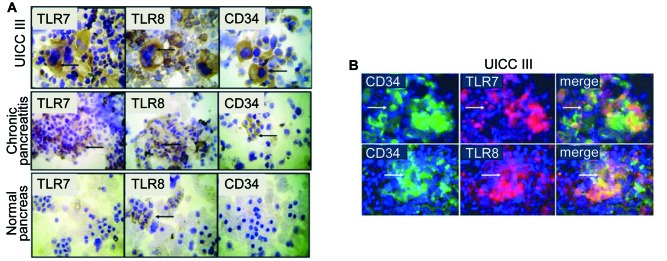
Immunohistochemical detection of TLR7 and TLR8 on cytospin samples of dissociated pancreatic cancer cells. (A) Increased expression of TLR7, TLR8 and CD34 was detected (arrows) in pancreatic cancer cells (UICC III). Elevated levels of TLR7 and TLR8 were also found in pancreatic cells from chronic pancreatitis while CD34 was not or sporadic detectable (arrows). No positivity of TLR7 and CD34 was detected in normal pancreatic cells, little expression of TLR8 was observed (arrow). DAB (3,3′-diaminobenzidine) brown color, Haemalaun blue color for nuclear counterstaining. (B) Immunofluorescence double staining in pancreatic cancer cells (UICC III, cytospins) showed increased coexpression (merge) of CD34 (FITC green and DAPI blue for nuclear counterstaining) with TLR7 (Cy3 red and DAPI blue for nuclear counterstaining) and TLR8 (Cy3 red and DAPI blue for nuclear counterstaining) (arrows). FITC, fluorescein isothiocyanate; DAPI, 4′,6-Diamidino-2-phenylindoledihydrochloride.

Furthermore, we analyzed TLR7 and TLR8 in dissociated cells derived from the same patient tissues together with CD34, a marker for endothelial cells and known to be expressed by cancer cells with neoangiogenetic potential, by FACS and immunohistochemical analysis (cytospins). Indeed TLR7, TLR8 and CD34 were positively expressed in pancreatic cancer and pancreatic cells from chronic pancreatitis cells (Figs. 2B and C and 3A), but not or at very low levels in normal pancreatic cells (Figs. 2A and 3A). Comparison of the cellular co-localization of TLR7 or TLR8 with CD34 analyzed by immunofluorescence double staining revealed increased coexpression of TLR7 or TLR8 with CD34 in tumor cells (Fig. 3B), indicating that those cells were indeed cancer cells expressing the angiogenic surface molecule.

### COX-2 is expressed in pancreatic cancer cells

**Figure 4:**
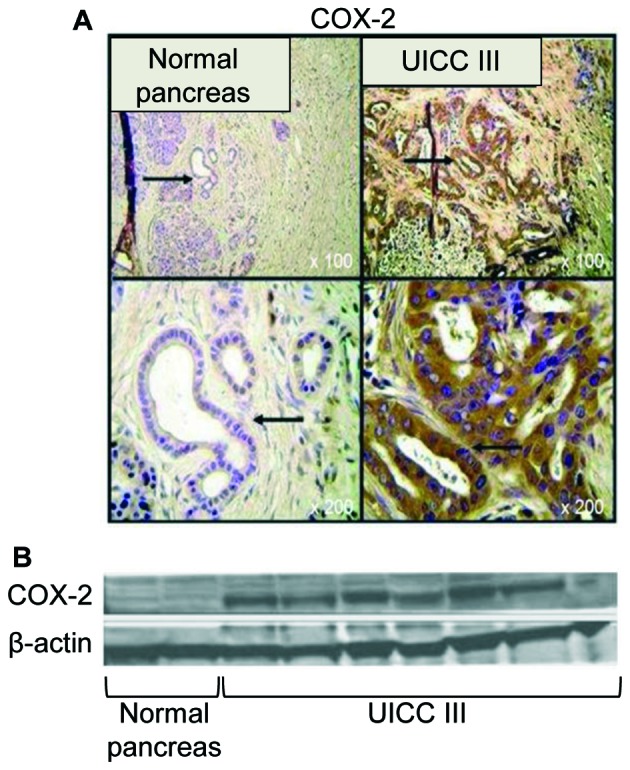
Detection of COX-2 expression in pancreatic cancer and normal pancreatic tissue. (A) Increased COX-2 expression in pancreatic cancer compared to normal tissue was detected in immunohistochemical staining (arrows). DAB (3,3′-diaminobenzidine) brown color, Haemalaun blue color for nuclear counterstaining. (B) Confirmation of increased COX-2 expression in pancreatic cancer (UICC III) compared to normal tissue by western blot analysis. β-actin probe was used as a control for protein loading.

To analyze whether inflammation in pancreatic cancer was associated with TLR7 and TLR8 expressing cancer cells, we dissected the expression of COX-2 in the pancreatic tumor cells by immunohistochemical staining and western blot analysis. Increased COX-2 expression together with TLR7 and TLR8 positivity in pancreatic cancer cells was detected (Fig. 4A, top and below right, and B, respectively). No positivity was observed in normal pancreatic cells (Fig. 4A, top and below left, and B, respectively). These data demonstrate inflammation in pancreatic cancer in association with TLR7 and TLR8 expressing cancer cells.

### TLR7 and TLR8 are expressed by human pancreatic cancer cell lines

**Figure 5:**
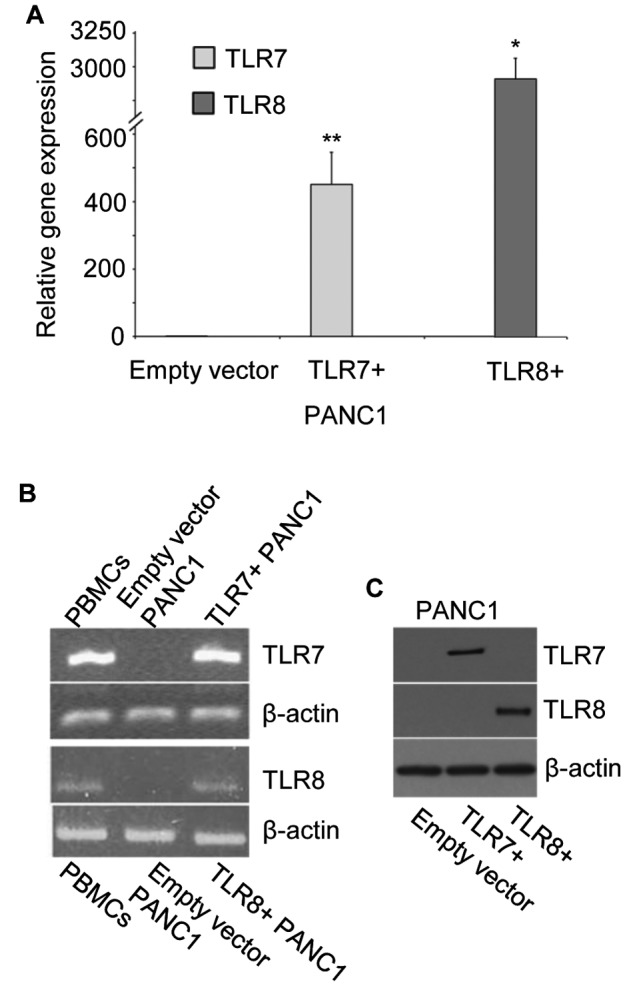
Depiction of the successful transduction of TLR7 and TLR8 in PANC 1 cells. (A) Increased gene expression of TLR7 and TLR8 was detected in TLR7^+^ and TLR8^+^ PANC1 cells by RT-qPCR compared to empty vector PANC1 cells. Empty vector PANC1 cells were standardized to baseline. The relative gene expression is expressed as 2^−ΔΔCq^; ^*^P<0.0001, ^**^P<0.005. (B) Agarose gel electrophoresis of RT-qPCR products. PANC1 cells transduced with empty vector and peripheral blood mononuclear cells (PBMCs) were used as controls. β-actin probe was used as internal control for RT-qPCR. (C) Confirmation of increased TLR7 and TLR8 protein expression in transduced PANC1 cells by western blot analysis. PANC1 cells transduced with empty vector were used as controls. β-actin probe was used as a control for protein loading.

We characterized the expression of TLR7 and TLR8 in several purchased human pancreatic cancer cell lines. In contrast to tumor cells derived from our patients with pancreatic cancer, acquired tumor cell lines expressed only very low levels of TLR7 and TLR8. This may be due to artificial, non-inflammatory culture conditions of the cell lines. Therefore, for further *in vitro* studies both receptors were successfully transduced in the most common pancreatic cell line, PANC1, using a Lentivirus-mediated stable gene expression as described in Materials and methods. As controls, PANC1 cells transduced with empty vector construct as well as peripheral blood mono-nuclear cells (PBMCs), were used. Indeed, increased gene expression of TLR7 and TLR8 was observed in the transduced PANC1 cells (TLR7^+^ and TLR8^+^ PANC1 cells) by qRT-PCR and following agarose gel electrophoresis (Fig. 5A and B). In Fig. 5C successful protein expression of TLR7 or TLR8 by transduced PANC1 cells was demonstrated by western blot analysis.

### TLR7 and TLR8 expression increases tumor growth in Balb/c nude mice

**Figure 6:**
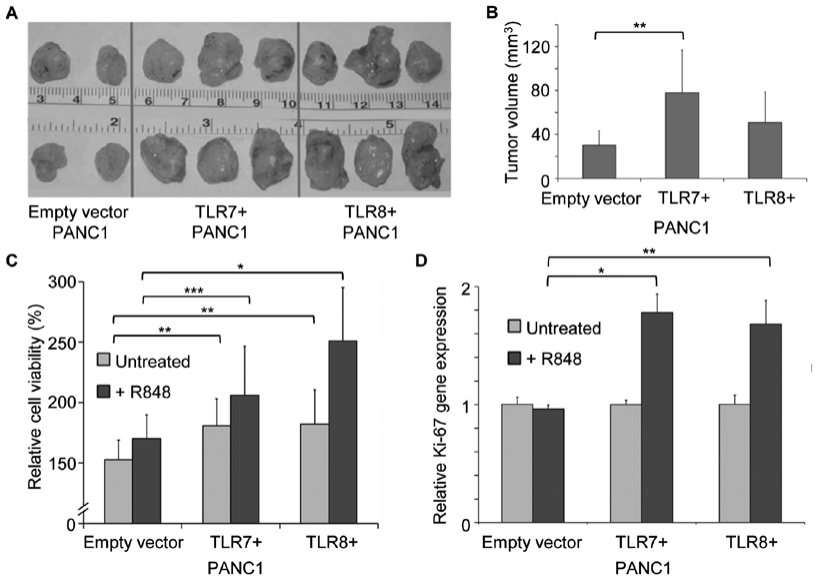
Expression and stimulation of TLR7 and TLR8 causes increased proliferation in TLR7^+^ and TLR8^+^ PANC1 cells. (A) Increased tumor size in subcutaneously injected Balb/c nude mice triggered by TLR7^+^ (n=5) and TLR8^+^ (n=5) PANC1 cells compared to empty vector PANC1 cells (n=4). (B) Significant increase in tumor volume caused by TLR7^+^ PANC1 cells in Balb/c nude mice compared to empty vector cells (^**^P<0.005). (C) Significantly accelerated proliferation of TLR7^+^ and TLR8^+^ PANC1 cells without (^**^P<0.002 and ^**^P<0.005) and with R848 stimulation (^***^P<0.02 and ^*^P<0.0001) compared to empty vector PANC1 cells analyzed by MTS assay. (D) Increased gene expression of Ki-67 in R848 stimulated TLR7^+^ and TLR8^+^ PANC1 (^*^P<0.0001 and ^**^P<0.0005) cells compared to empty vector PANC1 cells. Data of three independent experiments are shown with standard deviation. Untreated PANC1 cells were standardized to baseline. The relative gene expression is expressed as 2^−ΔΔCq^.

Tumor xenograft growth of TLR7 and TLR8 transduced human PANC1 cancer cells in Balb/c nude mice was examined. Tumor growth *in vivo* was found to be enhanced when compared to controls with empty vector PANC1 cells (Fig. 6A; TLR7^+^ and TLR8^+^, each n=5 vs. empty vector, n=4). Determination of the tumor growth showed a significant increase in tumor volume of TLR7^+^ PANC1 pancreatic tumors in contrast to empty vector PANC1 tumors (Fig. 6B; P<0.005).

### TLR7 and TLR8 expression and stimulation induces proliferation of PANC1 cells

The promoting effect of TLR7 and TLR8 expression on PANC1 cancer cell proliferation was analyzed using MTS proliferation assays. Untreated TLR7^+^ and TLR8^+^ PANC1 cells showed significantly increased tumor cell proliferation when compared to controls at 72 h after seeding (Fig. 6C; TLR7, 181% and TLR8, 182% vs. empty vector, 153%; P<0.002 and P<0.005).

We examined whether TLR7 and TLR8 stimulation with the agonist R848 further increases proliferation of TLR7^+^ and TLR8^+^ PANC1 cancer cells. Stimulation with the TLR7/TLR8 ligand R848 induced a relative increase in proliferation in TLR7^+^ and TLR8^+^ in PANC1 cancer cells compared to empty vector treated PANC1 cells (Fig. 6C; TLR7^+^, 206% and TLR8, 251% vs. empty vector, 170%; P<0.02 and P<0.0001). Gene expression of the proliferation marker Ki-67 in R848 treated TLR7^+^ and TLR8^+^ PANC1 cancer cells confirmed these proliferative effects. (Fig. 6D; P<0.0001 and P<0.0005).

### TLR7 or TLR8 stimulation of human PANC1 cells induces gene expression of NF-κB and COX-2

**Figure 7:**
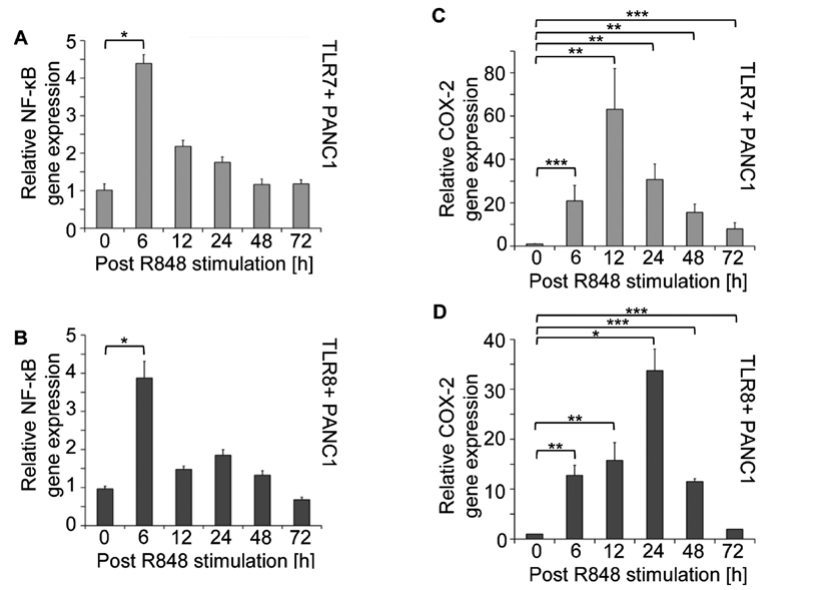
NF-κB and COX-2 gene expression in response to R848 stimulation of TLR7^+^ and TLR8^+^ PANC1 cells. (A and B) Stimulation of TLR7^+^ and TLR8^+^ PANC1 cells with R848 resulted in significantly increased gene expression levels of NF-κB (^*^P<0.0001) 6 h post stimulation. (C and D) Significantly escalated COX-2 gene expression levels 6–72 h after stimulation with maximum expression for TLR7^+^ PANC1 cells at 12 h (^**^P<0.005, ^***^P<0.05) and for TLR8^+^ PANC1 cells at 24 h (^*^P<0.0001, ^**^P<0.005, ^***^P<0.05). Data of three independent experiments are shown with standard deviation. Untreated PANC1 cells were standardized to baseline. The relative gene expression is expressed as 2^−ΔΔCq^.

To determine whether TLR7 and TLR8 stimulation activates intracellular signaling pathways and the synthesis of proinflammatory cytokines, we analyzed gene expression levels of NF-κB and COX-2 in response to stimulation of TLR7^+^ and TLR8^+^ PANC1 cells with R848. Stimulation with R848 for 6 h induced an ~4-fold increase in gene expression levels of NF-κB in TLR7^+^ and TLR8^+^ PANC1 cancer cells compared with untreated cells (Fig. 7A and B; P<0.0001). Seventy-two hours after stimulation with R848 NF-κB expression returned to background levels in both TLR7^+^ and TLR8^+^ PANC1 cancer cells. Additionally, stimulation with R848 induced an ~60-fold increased gene expression of COX-2 in TLR7^+^ PANC1 cancer cells (12 h after stimulation, Fig. 7C) and an ~34-fold increased level in TLR8^+^ PANC1 cells (24 h after stimulation, Fig. 7D) compared with untreated cells (Fig. 7C and D; P<0.005 and 0.0001). Even 72 h post-stimulation COX-2 expression levels remained significantly elevated in stimulated TLR7^+^ and TLR8^+^ cancer cells in comparison to untreated cancer cells.

### TLR7 or TLR8 stimulation induces chemoresistance in PANC1 cells

**Figure 8:**
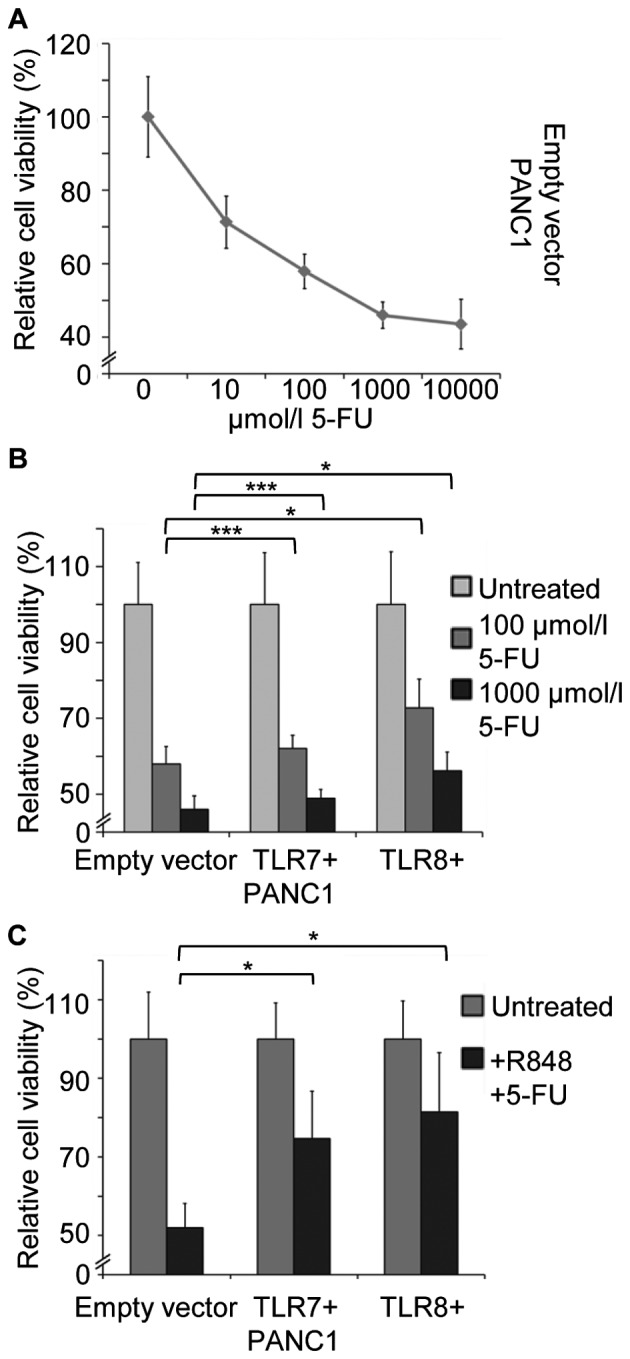
Effect of 5-fluorouracil on TLR7^+^ and TLR8^+^ PANC1 cells. (A) LD_50_ concentration of 5-fluorouracil (5-FU) for empty vector PANC1 cells was found at a concentration of 500 μmol/l by MTS assay. (B) Decreased sensitivity of TLR7^+^ PANC1 cells (^***^P<0.05) and TLR8^+^ PANC1 cells (^*^P<0.0001) to different concentrations of 5-FU compared to empty vector PANC1 cells analyzed by MTS assay. Untreated PANC1 cells were standardized to baseline. (C) Significantly reduced chemosensitivity of R848 and 5-FU (500 μmol/l) treated TLR7^+^ PANC1 cells and TLR8^+^ PANC1 cells compared to empty vector PANC1 cells (^*^P<0.0001) found by MTS assay. Untreated PANC1 cells were standardized to baseline.

To analyze the influence on chemoresistance in R848 stimulated and non-stimulated TLR7^+^ and TLR8^+^ PANC1 cancer cells 5-fluorouracil was used. 5-FU is amongst other chemotherapeutics used as treatment for pancreatic cancer (18) and thus herein used as representative chemotherapeutic agent. We first determined the LD_50_ concentration for 5-FU (500 μmol/l) using non-stimulated empty vector PANC1 cells in MTS assays (Fig. 8A).

To investigate the effects of induced TLR7 and TLR8 expression in PANC1 cancer cells on chemosensitivity transduced tumor cells were treated with two different concentrations of 5-FU (100 and 1000 μmol/l) as approximated concentrations for LD_50_. For both concentrations increased cell viability of TLR7^+^ and TLR8^+^ PANC1 cancer cells was demonstrated when compared to empty vector PANC1 cells, pointing to an increased chemoresistance in the cells. At a concentration of 100 μmol/l of 5-FU relative cell viability of TLR7^+^ and TLR8^+^ PANC1 tumor cells was less reduced when compared with empty vector PANC1 tumor cells (Fig. 8B; 62 and 73% vs. 58% for empty vector cells; P<0.05 and P<0.0001). This effect was confirmed at a concentration of 1000 μmol/l of 5-FU (TLR7^+^ and TLR8^+^ cells, 49 and 56% vs. 46% in empty vector cells (Fig. 8B; P<0.05 and P<0.0001).

Stimulation of TLR7^+^ and TLR8^+^ PANC1 cancer cells for 48 h with the agonist R848 prior to treatment with 500 μmol/l of 5-FU (LD_50_ for empty vector PANC1 cells) increased cell viability of TLR7^+^ and TLR8^+^ cells in contrast to empty vector PANC1 cells (Fig. 8C; TLR7^+^, 75% and TLR8^+^, 81% vs. empty vector PANC1 cells, 52%; both P<0.0001).

## Discussion

We previously reported that TLR7 and TLR8 expression is upregulated in tumor cells of patients with colorectal cancer. Interestingly, this expression was related to cancer cells but rarely detected in stromal-tumor-infiltrating leukocytes. Moreover, our results indicated that both TLR7 and TLR8 expression is associated with tumor progression in patients with colorectal cancer and reduced tumor-specific survival among patients with high TLR7 and TLR8 expression in their cancer cells (13).

In the present study, we demonstrated that TLR7 and TLR8 expression are highly expressed by primary human ductal pancreatic cancer. We showed that stimulation of both receptors TLR7 and TLR8 in pancreatic cancer cells results in increased tumor cell proliferation and reduced chemosensitivity.

To analyze the impact of the intracellular TLR7 and TLR8 expression in mediating inflammation in pancreatic cancer cells we first examined in the present study their expression in human tissues from primary pancreatic cancers. We observed that tumor cells in pancreatic cancer strongly expressed stage-dependent TLR7 and TLR8. This was intensified when compared to pancreatic cells in chronic pancreatitis. Whether intracellular TLR7 and TLR8 expression, known to be associated with single stranded RNA (virus) infection in this context may be associated with recognition of pathogenic viruses in the investigated human pancreatic cancers remains speculative. In our investigated human pancreatic cancers we did not find any evidence from medical records or from virus genome analysis. These data suggest that inflammation within the tumor tissues could be mediated through TLR7 and TLR8 expressing pancreatic cancer cells. Thus, intracellular TLR7 and TLR8 signaling pathways in TLR7^+^ and TLR8^+^ expressing pancreatic cancer cells may have the potential to sustain cancer progression. CD34 is a known marker for endothelial cells and is expressed by cancer cells with neoangiogenetic potential. Cell morphology of cancer cells and positive staining for CD34 indicated that cells expressing TLR7 and TLR8 were indeed cancer cells.

Invasion and angiogenesis of gastric cancer cells was described to be mediated by cyclooxygenase-2 (COX-2) after TLR2 and TLR9 activation, leading to inflammation and cancer progression (19). Moreover, increased COX-2 expression in human pancreatic carcinomas supports the suggestion that these tumors share common features of chronic inflammatory processes in parallel to all essential features of carcinogenesis (mutagenesis, mitogenesis, angiogenesis, reduced apoptosis, metastasis and immunosuppression). All these events are linked to COX-2-driven prostaglandin (PGE-2) biosynthesis (20–22). TLR8 signaling was recently described to strongly promote inflammatory lipid mediator biosynthesis PGE2 and thromboxane A2 (TXA2) through the COX-2 pathway. These data provide novel insights into the innate immune response to viral infections and raise the possibility that the immune response to single-stranded RNA viruses via the TLR8 pathway may implicate the lipid mediators of inflammation (23).

Notably, COX-2 expression was indeed upregulated in our investigated patient tumors and was associated with TLR7 and TLR8 positivity in specimens of pancreatic cancer and after stimulation of human PANC1 cancer cells. These data clearly indicate that inflammation in pancreatic cancer is associated stage-dependently with upregulated TLR7 and TLR8 expression in the cancer cells. Moreover, TLR7 and TLR8 stimulation in human PANC1 cancer cells led to the release of inflammatory mediators, mainly through the activation of the NF-κB pathway. It is known, that pancreatic carcinogenesis is attributed to the deregulated expression of many signaling elements, such as NF-κB. This signaling pathway leads to activation of mitotic and survival pathways (Bcl-2, bcl-XL), as it was described for EGF-EGFR signaling (24). In our so far unpublished preliminary data studying inflammatory cells and tumor cells within the tumor microenvironment in pancreatic cancer resulted from SABiosciences RT2 pathway array analysis, we observed strong regulation of several genes. This includes Bcl-2 in PANC1 pancreatic cancer cells stimulated with an agonist for TLR7 and TLR8. We also found in response to TLR7 and TLR8 stimulation an upregulation of several genes involved in angiogenesis as well as proinflammatory cytokines such as IL-8 and IL-12. Further studies are needed to confirm these first data.

Chemotherapy is a conventional regimen for unresectable cases of pancreatic cancer. However, treatment with chemotherapy drugs, like 5-FU or gemcitabine, merely results in a median survival of 5.65 months and 1-year survival rate of 18% (25). The main reason for chemotherapy failure lies in the intrinsic and acquired chemoresistance of pancreatic cancer cells (26). Recent data pointed to the role of the Notch-2 receptor in the increasing of chemoresistance in the pancreatic cancer (27). TLR7 and TLR8 seems to stimulate the expression of Notch-2 receptor (28). It seems that there is a link between TLR7 and TLR8 expression and the activation of Notch. Notably, stimulation of TLR7 and TLR8 in the present study also resulted in a more robust chemoresistance in PANC1 cancer cells against 5-fluorouracil. Further studies must be performed to confirm our hypothesis.

To date, several agonists have been characterized as TLR7 and/or TLR8 ligands. Resiquimod (R848) exerts its immunostimulatory activities via activation of mouse TLR7 and human TLR7 and TLR8 (29,30). The agonist R848 has now also been tested as an immune response modifier in preclinical models and in clinical trials (31,32). It has been shown that TLR agonists can promote cancer cell survival and migration and tumor progression. For example, TLR agonists have been shown to increase tumor viability and metastasis of human lung cancer cells (10), proliferation in human myeloma cells (TLR3) (33), adhesion and metastasis in human colorectal cancer cells (TLR4) (34), and migration in human gliobastoma (TLR4) or human breast cancer cells (TLR2) (35). We hypothesized that these contradictory results are due to the complex nature of the tumor microenvironment. Interestingly, in the present study in pancreatic cancer we observed that TLR7 or TLR8 stimulation increased tumor cell survival and resistance to the chemotherapeutic substance 5-fluorouracil. Further studies are necessary to dissect which cells and pathways are involved in these effects.

We conclude that inflammation-mediated progression, tumor survival, metastatic potential and mediation of chemoresistance are closely associated with TLR7 and TLR8 expressing pancreatic cancer cells. Therefore, targeting of TLR signaling might be a potential mechanism to reduce chemoresistance, tumor surveillance and COX-2 induced carcinogenesis. However, the direct effects of immune response modifiers on tumor cells include induction of apoptosis and sensitization to killing mediated by chemotherapeutic agents. On the other hand, TLR activation can be advantageous for the proliferation, invasiveness, and/or survival of tumor cells. These effects of TLR7 and TLR8 agonists on tumor cells depend on the tumor cell type, and need to be carefully taken into account in preclinical studies.

## Abbreviations

DAMPs: damage-associated molecular patterns
COX-2: cyclooxygenase-2
NF-κB: nuclear factor kappa-light-chain-enhancer of activated B cells
PAMPs: pathogen-associated molecular patterns
PRR: pattern recognition receptor
UICC: Union International Contre le Cancer
TLR: Toll-like receptor
5-FU: 5-fluorouracil

## References

[1] Siegel R, Naishadham D, Jemal A (2013). Cancer statistics, 2013. CA Cancer J Clin.

[2] Saif MW (2007). Controversies in the adjuvant treatment of pancreatic adenocarcinoma. JOP.

[3] Saif MW (2011). Pancreatic neoplasm in 2011: An update. JOP.

[4] Balkwill F, Coussens LM (2004). Cancer: An inflammatory link. Nature.

[5] Coussens LM, Werb Z (2002). Inflammation and cancer. Nature.

[6] Greten FR, Karin M (2004). The IKK/NF-kappaB activation pathway-a target for prevention and treatment of cancer. Cancer Lett.

[7] Pikarsky E, Porat RM, Stein I, Abramovitch R, Amit S, Kasem S, Gutkovich-Pyest E, Urieli-Shoval S, Galun E, Ben-Neriah Y (2004). NF-kappaB functions as a tumour promoter in inflammation-associated cancer. Nature.

[8] Ketloy C, Engering A, Srichairatanakul U, Limsalakpetch A, Yongvanitchit K, Pichyangkul S, Ruxrungtham K (2008). Expression and function of Toll-like receptors on dendritic cells and other antigen presenting cells from non-human primates. Vet Immunol Immunopathol.

[9] Ochi A, Graffeo CS, Zambirinis CP, Rehman A, Hackman M, Fallon N, Barilla RM, Henning JR, Jamal M, Rao R (2012). Toll-like receptor 7 regulates pancreatic carcinogenesis in mice and humans. J Clin Invest.

[10] Cherfils-Vicini J, Platonova S, Gillard M, Laurans L, Validire P, Caliandro R, Magdeleinat P, Mami-Chouaib F, Dieu-Nosjean MC, Fridman WH (2010). Triggering of TLR7 and TLR8 expressed by human lung cancer cells induces cell survival and chemoresistance. J Clin Invest.

[11] Janeway CA (1989). Approaching the asymptote? Evolution and revolution in immunology. Cold Spring Harb Symp Quant Biol.

[12] Rubartelli A, Lotze MT (2007). Inside, outside, upside down: Damage-associated molecular-pattern molecules (DAMPs) and redox. Trends Immunol.

[13] Grimm M, Kim M, Rosenwald A, Heemann U, Germer CT, Waaga-Gasser AM, Gasser M (2010). Toll-like receptor (TLR) 7 and TLR8 expression on CD133^+^ cells in colorectal cancer points to a specific role for inflammation-induced TLRs in tumourigenesis and tumour progression. Eur J Cancer.

[14] Bowie A, O'Neill LA (2000). The interleukin-1 receptor/Toll-like receptor superfamily: Signal generators for pro-inflammatory interleukins and microbial products. J Leukoc Biol.

[15] Harris RE (2007). Cyclooxygenase-2 (cox-2) and the inflammogenesis of cancer. Subcell Biochem.

[16] Bong AB, Bonnekoh B, Franke I, Schön M, Ulrich J, Gollnick H (2002). Imiquimod, a topical immune response modifier, in the treatment of cutaneous metastases of malignant melanoma. Dermatology.

[17] Dunne A, Marshall NA, Mills KH (2011). TLR based therapeutics. Curr Opin Pharmacol.

[18] Conroy T, Desseigne F, Ychou M, Bouché O, Guimbaud R, Bécouarn Y, Adenis A, Raoul JL, Gourgou-Bourgade S, de la Fouchardière C, Groupe Tumeurs Digestives of Unicancer; PRODIGE Intergroup (2011). FOLFIRINOX versus gemcitabine for metastatic pancreatic cancer. N Engl J Med.

[19] Chang YJ, Wu MS, Lin JT, Chen CC (2005). Helicobacter pylori-induced invasion and angiogenesis of gastric cells is mediated by cyclooxygenase-2 induction through TLR2/TLR9 and promoter regulation. J Immunol.

[20] de Moraes E, Dar NA, de Moura Gallo CV, Hainaut P (2007). Crosstalks between cyclooxygenase-2 and tumor suppressor protein p53: Balancing life and death during inflammatory stress and carcinogenesis. Int J Cancer.

[21] Dvorak HF (2005). Angiogenesis: Update 2005. J Thromb Haemost.

[22] Yip-Schneider MT, Barnard DS, Billings SD, Cheng L, Heilman DK, Lin A, Marshall SJ, Crowell PL, Marshall MS, Sweeney CJ (2000). Cyclooxygenase-2 expression in human pancreatic adenocarcinomas. Carcinogenesis.

[23] Hattermann K, Picard S, Borgeat M, Leclerc P, Pouliot M, Borgeat P (2007). The Toll-like receptor 7/8-ligand resiquimod (R-848) primes human neutrophils for leukotriene B4, prostaglandin E2 and platelet-activating factor biosynthesis. FASEB J.

[24] Meteoglu I, Erdogdu IH, Meydan N, Erkus M, Barutca S (2008). NF-KappaB expression correlates with apoptosis and angiogenesis in clear cell renal cell carcinoma tissues. J Exp Clin Cancer Res.

[25] Burris HA, Moore MJ, Andersen J, Green MR, Rothenberg ML, Modiano MR, Cripps MC, Portenoy RK, Storniolo AM, Tarassoff P (1997). Improvements in survival and clinical benefit with gemcitabine as first-line therapy for patients with advanced pancreas cancer: A randomized trial. J Clin Oncol.

[26] Wang Z, Li Y, Ahmad A, Banerjee S, Azmi AS, Kong D, Sarkar FH (2011). Pancreatic cancer: Understanding and overcoming chemoresistance. Nat Rev Gastroenterol Hepatol.

[27] Güngör C, Zander H, Effenberger KE, Vashist YK, Kalinina T, Izbicki JR, Yekebas E, Bockhorn M (2011). Notch signaling activated by replication stress-induced expression of midkine drives epithelial-mesenchymal transition and chemoresistance in pancreatic cancer. Cancer Res.

[28] Hu X, Chung AY, Wu I, Foldi J, Chen J, Ji JD, Tateya T, Kang YJ, Han J, Gessler M (2008). Integrated regulation of Toll-like receptor responses by Notch and interferon-gamma pathways. Immunity.

[29] Hemmi H, Noike M, Nakayama T, Nishino T (2002). Change of product specificity of hexaprenyl diphosphate synthase from Sulfolobus solfataricus by introducing mimetic mutations. Biochem Biophys Res Commun.

[30] Jurk M, Heil F, Vollmer J, Schetter C, Krieg AM, Wagner H, Lipford G, Bauer S (2002). Human TLR7 or TLR8 independently confer responsiveness to the antiviral compound R-848. Nat Immunol.

[31] Scheel B, Aulwurm S, Probst J, Stitz L, Hoerr I, Rammensee HG, Weller M, Pascolo S (2006). Therapeutic anti-tumor immunity triggered by injections of immunostimulating single-stranded RNA. Eur J Immunol.

[32] Sauder DN, Smith MH, Senta-McMillian T, Soria I, Meng TC (2003). Randomized, single-blind, placebo-controlled study of topical application of the immune response modulator resiquimod in healthy adults. Antimicrob Agents Chemother.

[33] Chiron D, Pellat-Deceunynck C, Amiot M, Bataille R, Jego G (2009). TLR3 ligand induces NF-{kappa}B activation and various fates of multiple myeloma cells depending on IFN-{alpha} production. J Immunol.

[34] Hsu RY, Chan CH, Spicer JD, Rousseau MC, Giannias B, Rousseau S, Ferri LE (2011). LPS-induced TLR4 signaling in human colorectal cancer cells increases beta1 integrin-mediated cell adhesion and liver metastasis. Cancer Res.

[35] Thuringer D, Hammann A, Benikhlef N, Fourmaux E, Bouchot A, Wettstein G, Solary E, Garrido C (2011). Transactivation of the epidermal growth factor receptor by heat shock protein 90 via Toll-like receptor 4 contributes to the migration of glioblastoma cells. J Biol Chem.

